# A review of higher-order mode pass filtering techniques

**DOI:** 10.1016/j.heliyon.2022.e11705

**Published:** 2022-11-18

**Authors:** Prapty Saha, M. Salauddin Rasel, Kazi Tanvir Ahmmed

**Affiliations:** a Department of Electrical and Electronic Engineering, University of Chittagong, Chittagong, 4331, Bangladesh; b Department of Electrical and Electronic Engineering, East Delta University, Chittagong, 4209, Bangladesh

**Keywords:** Mode filter, Mode converter, Planar, Polymer waveguide, Long-period fiber gratings

## Abstract

Mode Division Multiplexing (MDM) is regarded as a promising technology to overcome the bottleneck of the future demand for high data transmission rates. Multimode fibers are replacing traditional single mode fibers to cope with the increasing bandwidth requirements. MDM works with multiple light modes, and these modes are bound to be coupled with other propagating modes as they pass through guided media. A mode filter removes unwanted modes from the signal received at the receiver after demultiplexing. As a result, designing a highly potential high-order mode pass filter is desired to meet the capacity crunch using the MDM technology. Currently, remarkable research works have been conducted on mode filtering. This paper presents an overview of recent developments in mode filtering techniques along with their designs and fabrication processes. In particular, the mode filter made from different types of materials is reviewed to illustrate the potential of the designs in improving the system performance. Even though commercial success has not been materialized, this work will provide promising prospects in mode filtering techniques for increased flexibility and the choice of different mode filters.

## 1 Introduction

Network traffic has grown exponentially over decades and the services provided to the users are not enough to fulfill the increasing demand for those services. This causes a capacity crunch. The single-mode fiber has a maximum transmission capacity of 100 Tbit/s which is not adequate for the required demands. Such challenges lead to finding out ingenious ways by introducing different multiplexing systems like wavelength-division multiplexing (WDM), polarization-division multiplexing (PDM), space-division multiplexing (SDM), and so on. Further researches find out that these techniques have limitations due to the physical limits or design complexities. Currently, multi-mode and few-mode fiber has attracted the attention of the researchers in the field of optical fiber communication to solve the transmission problem which establishes the mode-division multiplexing (MDM) technique [1]. In the MDM process, each mode works as an independent transmission channel, and by permitting high-order mode this process increases the efficiency of the transmission links. After using demultiplexer the undesired modes can be filtered out by the use of mode filter. Thus, the term mode filtering has been introduced in this field to make the transmission process more flexible. In recent years, silicon photonics in both SDM and MDM systems has gained significant research interest due to its cost efficiency and CMOS competent fabrication process without compromising its high-performance capability [2, 3, 4]. A planar lightwave circuit (PLC) is one of the most preferred platforms for realizing a compact MDM process for achieving high packing density in a photonic integrated circuit. Various MDM devices such as mode multiplexers, demultiplexers [5, 6], mode converters [7, 8], mode selective switches [9, 10, 11], mode splitter [12] have already been demonstrated on the SOI platform. Because of restricted performance, non-trivial modal crosstalk, narrow bandwidth, and sensitivity to manufacturing traditional demultiplexers are not used as a mode filter. In on-chip MDM, the mode filter has become an indispensable element that works similarly to the wavelength filter in WDM [2]. Mode filters are critical in MDM systems for filtering unwanted modes after demultiplexing different optical modes. Furthermore, spatial mode filters are required in the fibre span to suppress unwanted modes. Mode filters can reduce modal cross-talk and improve system performance significantly. High-order modes have weak optical confinement and can be easily stripped out with a tapered structure with a desirable mode cut-off width. Low-order modes, on the other hand, are difficult to filter out in the conventional structure due to their strong optical confinement in the core. A limited number of research works have been held in the development of the mode filtering technique. Hence, this field has dragged the attention of researchers and new possibilities are waiting to unfold.

In this paper, we are presenting a review of the recent progress and techniques introduced in this field within the last few years so that it can help the researchers of this field. We have summarized all the techniques along with their results in a table. Each structure explores different materials and different methodologies to establish the most prominent structure with high scalability, high flexibility and high extinction ratio (ER). When summarizing, we have analyzed all the uniqueness and weaknesses of each different structure. Finally, we believe that this work will help future researchers to understand the importance and challenges of this field and their contributions to developing a mature industry.

## 2 Types of mode filtering

Mode filtering is the process of selecting or rejecting modes or a range of modes. This process uses a mode filter for transmission or rejection of the modes. Mode filters can be used in a variety of applications for example life science, defence industries, clinical chemistry, and so on. There are two types of mode filters:

1. Lower-order mode (LOM) pass filter
2. Higher-order mode (HOM) pass filter

A lower-order mode filter passes the lower optical modes and blocks the higher-order modes. On the contrary, a higher-order mode filter passes the higher-order modes and blocks the lower-order modes. Mode filters can be utilized in many applications such as rejection of unwanted signals in a telecommunication system, tuning the modes, balancing the response of photo-detector, and so on. The filters can be also used as a bandpass filter where a defined range of modes can be filtered out.

## 3 Mode filtering techniques

### 3.1 Mode filter based on optically resonant device

Optical Resonant Devices exhibit resonant behaviour with varying wavelengths of light waves. Long-period grating (LPG), photonic crystals, SWGs are examples of optical resonant devices. Based on these devices several mode filtering techniques have been introduced.

A 1D photonic crystal grating silicon waveguide-based higher-order mode pass filter was experimentally demonstrated by Guan et al. [13] in 2015. It was designed in such a way that the high-order mode worked on the air gap and passed through the crystal with lower insertion loss converting into Bloch mode. Though the measured ER is 50dB, the ER varies up to ±10dB due to the presence of limitations in the setup of the detection mechanism. The setup mechanism can be studied for improving the performance of the device.

The long period grating is a structure that has a period typically in the range of 100 μm to 1mm. In 1996, Vengsarkar and co-workers first reported that LPG couples light from a principal guided center mode into co-proliferating cladding modes at different wavelengths [14]. The intermittent length of the LPG relies upon the neighbourhood condition, for example, temperature, strain, twist span, and the refractive file of the medium encompassing the fiber. In this manner, any of the adjustments in these parameters can alter the period of the LPG and differential refractive file of the center and cladding which brings about a change in the focal wavelength of the weakening band by altering the stage coordinating conditions for coupling to the cladding modes [15, 16].

**Figure 1:**
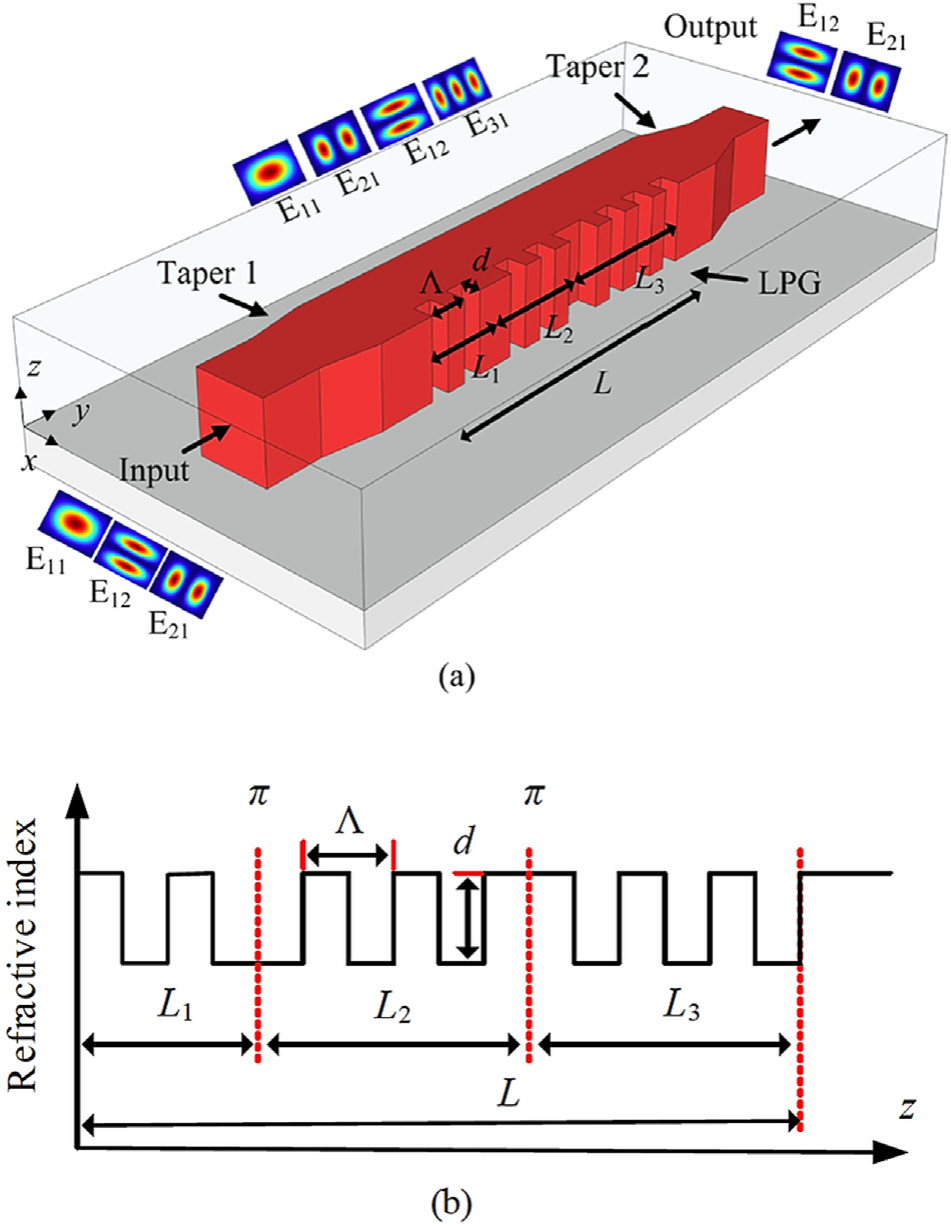
Schematic configuration of the a) proposed design and b) three sections of LPG, Reprinted with permission from [17] @ 2019 IEEE.

In 2019, Quandong Huang et al. [17] introduced an ultra-broadband mode filter which was a design of a few-mode waveguide with two linear tapers at two terminals and phase-shifted long-period grating at the middle. The proposed structure has three wave-guide sections connected by two identical linear tapers and a phase-shifted LPG formed along the middle section. Two ends of the waveguide of Figure 1 support E_11_, E_21_, and E_12_ modes. By optimizing the design E_11_ mode is converted into E_31_ mode and finally filtered out at the output end. Due to the absence of a selectively high-order modes excitation method, this experimental device is incapable of measuring directly the spectral loss induced in higher-order modes. Reduction of the spectral loss and increase of the operational bandwidth can direct to future research.

W. Jiang et al. [18] a simulation-based design has been proposed using a high-order mode pass filter using cascaded plasmonic bridged subwavelength gratings (BSWGs). In the design of the filter, Aluminium bridge along with the cascaded silicon was introduced which formed a cascaded plasmonic BSWGs structure. This structure was able to influence the fundamental mode significantly without affecting the first-order mode. As a result, the first-order mode can pass through the structure and due to mode-mismatch the fundamental mode is radiate out and absorbed. They use 4-cascaded BSWG for better performance. The increase in the number of cascaded mode filters increases the device length whereas bulk devices are not favoured in commercial use.

**Figure 2:**
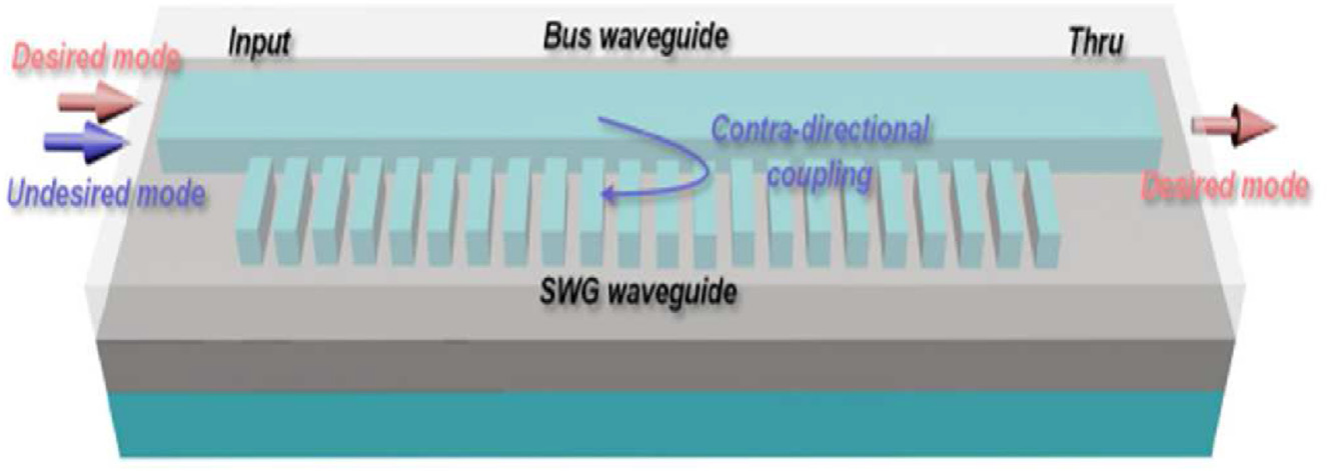
Structure of SWG based mode blocking filter, Reprinted with permission from [19] © The Optical Society.

In the field of radio technology, a directional coupler (DC) is a passive device used to couple a characterized measure of the electromagnetic power in a transmission line to a port empowering the signal to be utilized in another circuit. The main feature of DC is that only one-directional power is coupled. A silicon mode-blocking filter was introduced by Yu He et al. [19] using sub-wavelength grating (SWG) based contra directional coupler. This device was designed to block the unenvied mode channel without any impact on other modes of propagation. The design of Figure 2 has experimented which consists of an SWG waveguide along with a bus waveguide. The SWG waveguide is designed in such a way that the effective index of the selected mode meets the phase-matching condition of that waveguide and can be contra-directionally coupled to the Bloch mode. As a result, due to the phase mismatch other modes can go through the waveguide. To decrease the insertion loss of TE_02_ mode, the gap between two waveguides can be increased but this decreases the operation band of the device. This device also needs de-multiplexing stages separately which increases operational complexity.

### 3.2 Mode filter based on materials with special optical properties

In 2016, another mode filtering technique was proposed based on Hyperbolic Meta-Materials (HMM) as cladding [20]. The design of the waveguide consists of HMM as cladding and as the lower order modes have larger propagation constants, they become propagating waves in the HMM cladding turning into leaky modes. The high order modes with smaller propagation constant remain guided in the core due to being an evanescent wave in the HMM cladding. Thus, it worked as a mode selective high pass filter. As hyperbolic material is not available in nature for the wavelength of 1550nm, this result is analyzed only simulation-based.

**Figure 3:**
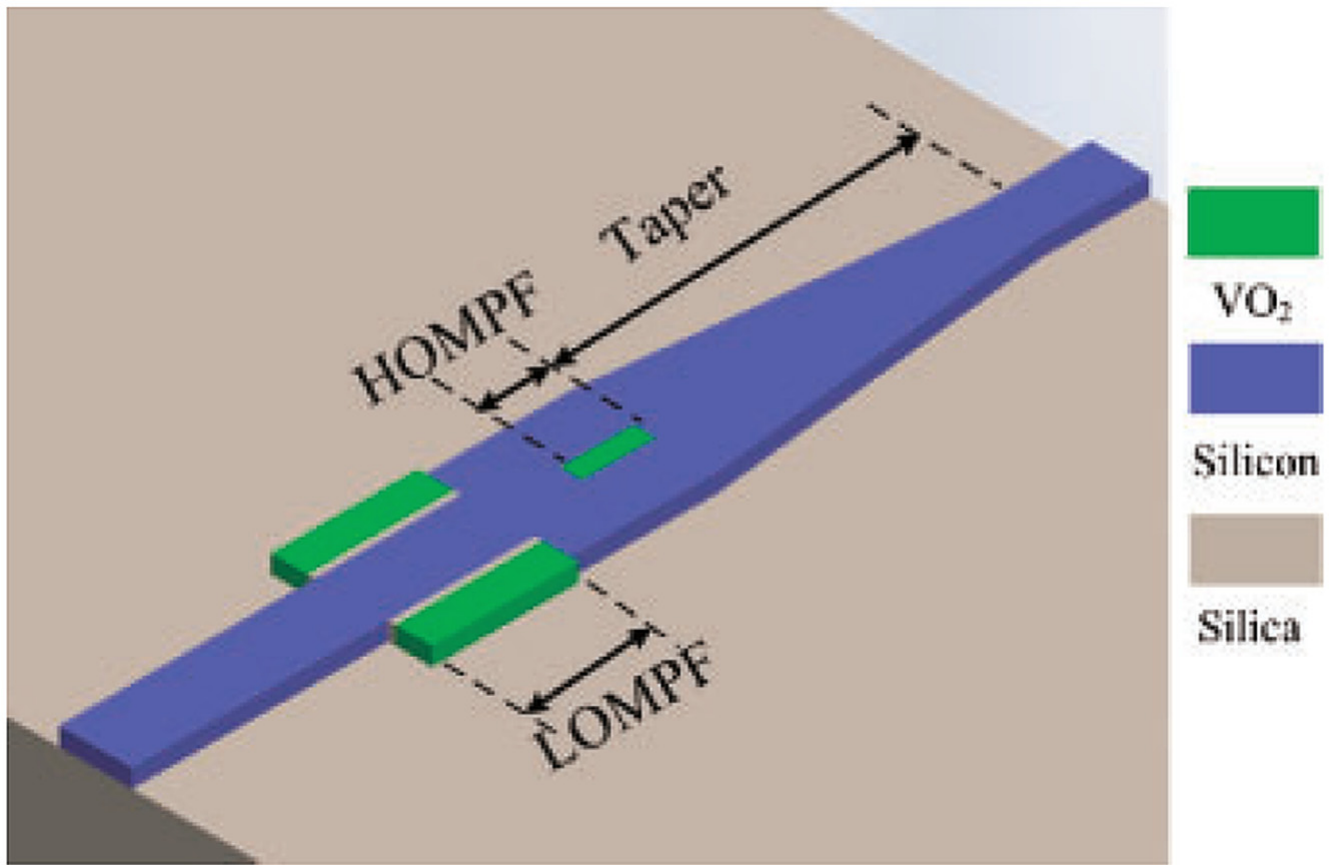
Schematic diagram of the reconfigurable mode filter [21] Copyright (2016) The Japan Society of Applied Physics.

A reconfigurable mode filtering technique was proposed by T. Huang et al. [21], 2016. This was a flexible approach based on phase transition in vanadium dioxide (VO2). The characteristics of VO2 vary depending on its working states i.e. steady-state and metallic state. Depending on these characteristics a waveguide was designed that supported two modes (TE_00_ and TE_01_) and worked as TE_00_ pass, TE_01_ pass, all pass, and all block individually. The simulated result shows that this device has low efficiency in filtering high-order mode. This technique is also limited due to the limited working states of the used material. Figure 3 is the cascaded configuration of the proposed structure.

In 2017, a study of graphene-embedded polymer waveguide was experimentally made which was limited in filtering either the fundamental transverse electric mode (TE) or the fundamental transverse magnetic mode (TM) [22]. To acknowledge mode channels by installing graphene at different locations into waveguides they showed a few common devices tentatively with varying lengths and widths. The experimental value of IL is ∼1 dB/cm larger compared to the theoretical value. This device has a limitation of blocking only the TE_0_ mode whereas the TM_0_ mode is allowed to pass.

P. Xing et al. [23] proposed a graphene-on-silicon waveguide mode filter for TE_0_ and TE_1_ mode. Graphene has the capability of absorbing light at a very broad spectrum. Based on this a simulated structure of the waveguide with the graphene layer was designed to absorb the unwanted mode. It was possible to adjust the intensity of the modal optical absorption by adjusting the graphene layer dimensions. The performance of the high-order mode filter is limited due to the dependency of absorption bandwidth on the spatial mode dispersion of that waveguide.

Guoquing You and Dingshan Gao proposed an ultra-compact higher-order mode filter based on a nonlinear direct binary search (DBS) optimization algorithm in 2019 [24] as an appealing algorithm for mode filtering technique. The design consists of an inverse design region with an ultra-compact footprint digitized into 13 Σ 20 pixels, an input waveguide, an adiabatic input taper, an output waveguide, and an adiabatic output taper. The states of each pixel can be changed and represented in '0′ or '1′ states. When light enters the waveguide the light wave is scattered and by optimizing the arrangement the designed mode filter only passes the first-order mode (TE_1_) while the fundamental mode (TE_0_) is deterred. However, this design is an only simulation based and the fabrication of an etched pixel with a radius of 45nm is a nontrivial process.

**Figure 4:**
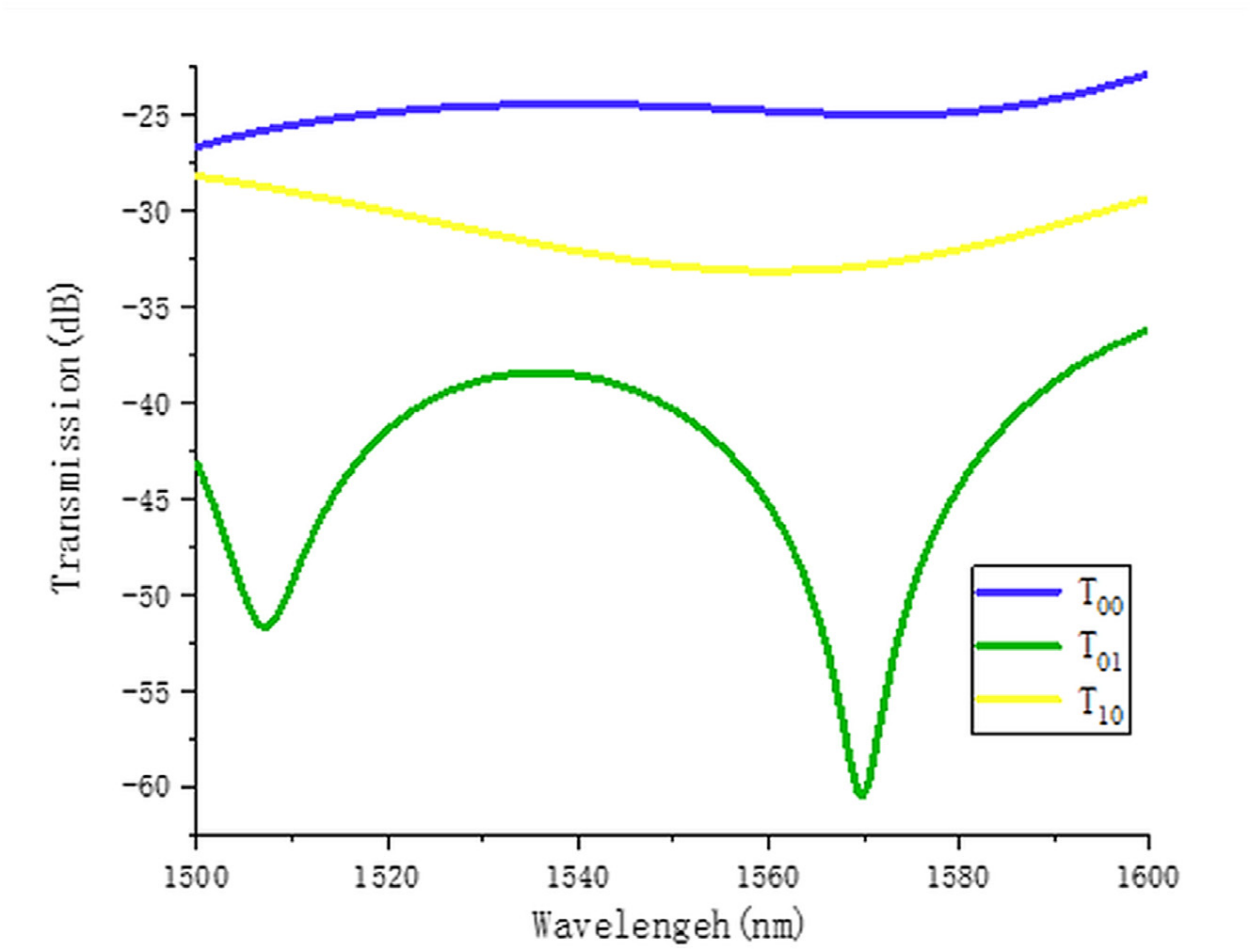
Simulated extinction ratio (ER) for the Inverse designed structure [24] Copyright (2019) The Japan Society of Applied Physics.

In Figure 4 the ER of the modes has been studied to analyze the performance of this technique. The simulated result is noticeable but fabrication is the ultimate challenge for realizing its performance experimentally.

Another graphene-based spatial mode filtering technique was proposed in 2019 by Z. Xing et al. [25]. In the simulated structure, graphene nanoribbons were used to filter out the lower-order modes. For TE_1_ -pass mode filter one nanoribbon was used and for TE_2_ –pass mode filter two nanoribbons were used. Both structures had different waveguide configurations with high optical losses. Fabricating this device on this scale is a challenging task.

### 3.3 Mode filter based on mode converter

When two or more beams are split from a single source, a phase shift occurs between the collimated beams due to the presence of a sample or a difference in path length. MZI is used for measuring the relative phase shift between these beams. In MZI, the wave which is passing through the arms is phase-shifted and when the two arms are recombined, the conversion of the wave into the amplitude modulation is carried out. MZI has a great application in designing Mux/de-Mux or reconfigurable Mux/de-Mux devices [5, 6].

Y-junction is a light-manipulating device which relies solely on geometrical design for its functionality [26]. Y-junction is wavelength-independent and consists of a stem and two diverging arms and its light splitting and light combing capabilities can be generalized to multi-arm Y-junctions [27, 28, 29]. Typically, these adiabatic devices can be subdivided into two categories: Symmetric and Asymmetric. The symmetric Y-junctions have the arms with the same cross-sections and same refractive indexes. In contrast to these, asymmetric Y-junctions have arms with different cross-sections and refractive indexes [30, 31].

**Figure 5:**
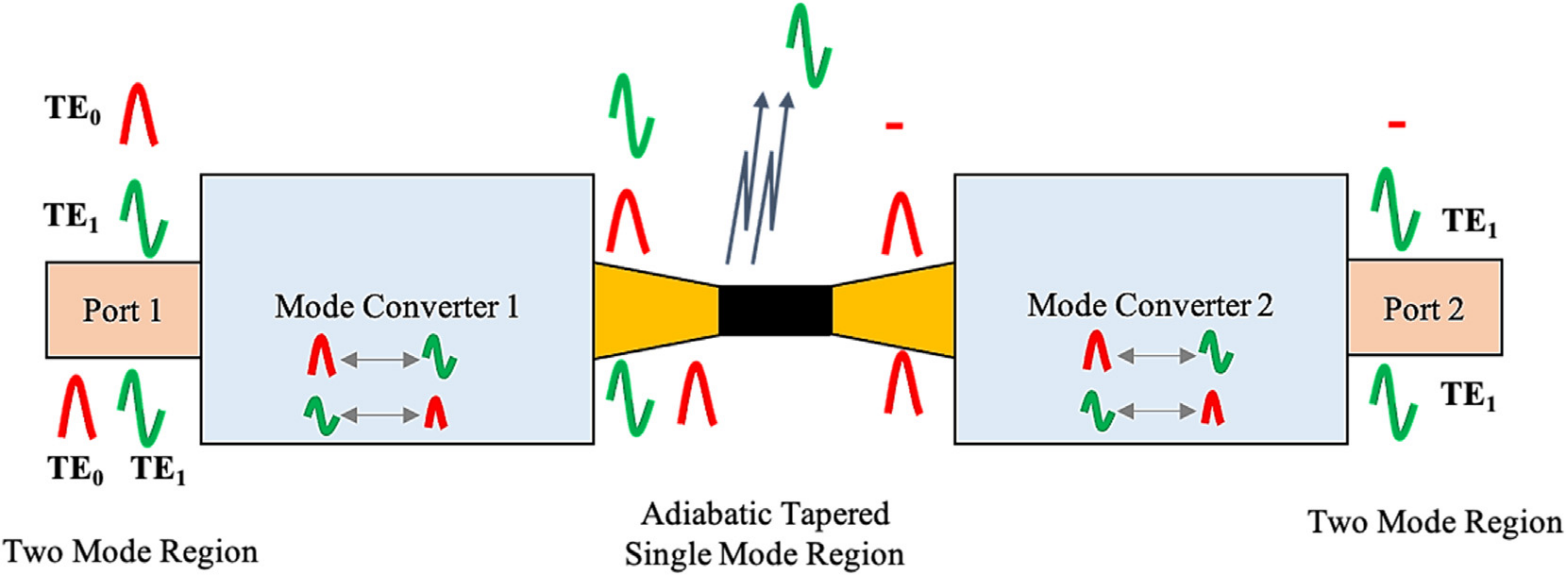
Schematic configuration of a mode converter-based two-mode, high-order mode-pass filter device, Reprinted with permission from [32] © The Optical Society.

Based on mode conversion in 2017, K. T. Ahmmed et al. [32] made a broadband high-order mode pass filter (Figure 5). The waveguide was designed using polymer materials and an adiabatic bi-conical tapered single-mode waveguide by placing it between two mode converter devices which were used for the conversion of the fundamental mode to the first-order mode and vice versa. Thence, the converted first-order mode can pass through the filter and be regained in the output port while the converted fundamental mode is blocked. This device offers a high extinction ratio and low optical loss. Like the MZI-based devices, this device is limited to phase conditions.

**Figure 6:**
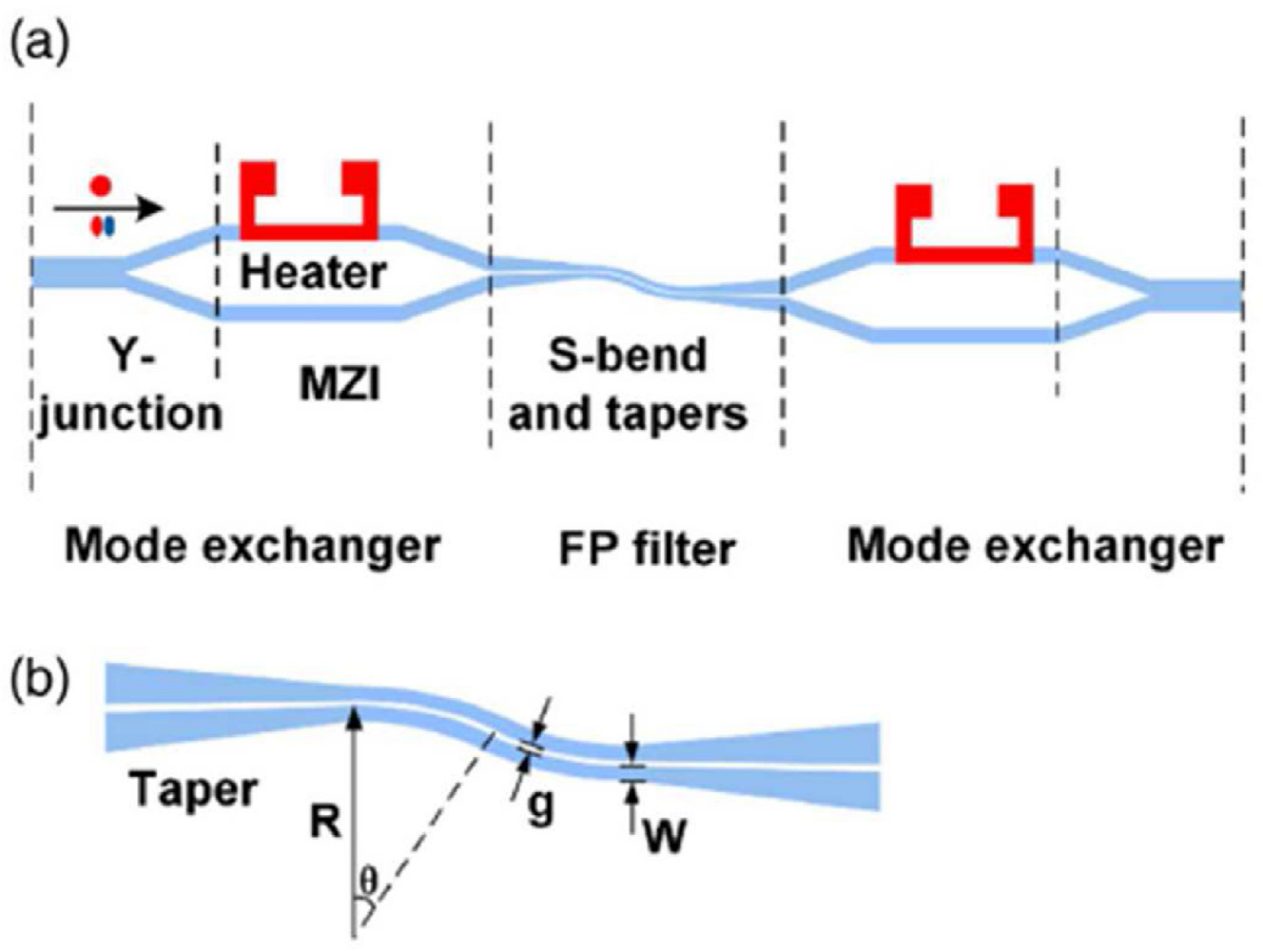
Schematic diagram of a) the device and b) the S-bend slot waveguide, Reprinted with permission from [33] © The Optical Society.

An integrated tunable mode filter for an MDM system was made at the beginning of 2018 [33] where the output mode can be tuned to the desired mode. This is also a mode exchanger-based design. The device in Figure 6 consists of one heater at the input MZI and another one at output MZI along with four Y-junctions. As a result, the device can be used both as a high pass filter and low pass filter, and at the same time, the output of the device can be tuned. This design works as an integrated tunable mode filter for the MDM system. This device is dependent on the phase conditions of the propagating modes. The basic idea of this design is similar to the design structure of [32], the only difference is the use of heaters in both MZI structures. Only the TE mode is studied here and the structure can be further studied for realizing TM modes. Though the IL and ER of this design are higher than the design of [32], the use of the heaters adds diversity to its operation by adding tuning capacity.

### 3.4 Mode filter based on multi-mode interference (MMI)

When a multimode waveguide is excited, the interference of the waves is produced due to the matching of the input fields and the modal fields within the waveguide. This effect is known as MMI [34]. MZI technology of different configurations can utilize MMI optical waveguide couplers and splitters. MMI can be used in mode filtering techniques because it has polarization properties, a higher bandwidth range, and manufacturing tolerance.

**Figure 7:**
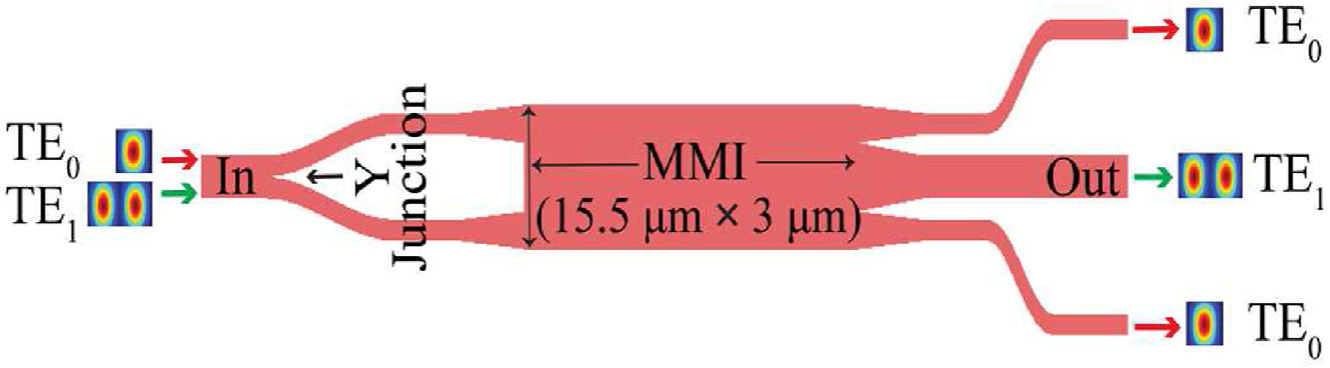
Schematic of TE_1_ pass TE_0_ filter device, redrawn from [35].

In a work of 2019, MMI and Y-junction based phase-insensitive higher-order mode-pass filter was proposed and experimentally demonstrated. The structure in Figure 7 was designed in such a way that the two in-phase TE_0_ modes will be obtained at the two outermost ports and the TE_1_ mode will be refocused at the central port. Thus the low-order modes will be filtered out [35]. Noise may be induced by accumulated reflection.

### 3.5 Mode filter based on asymmetric directional coupler (ADC)

**Figure 8:**
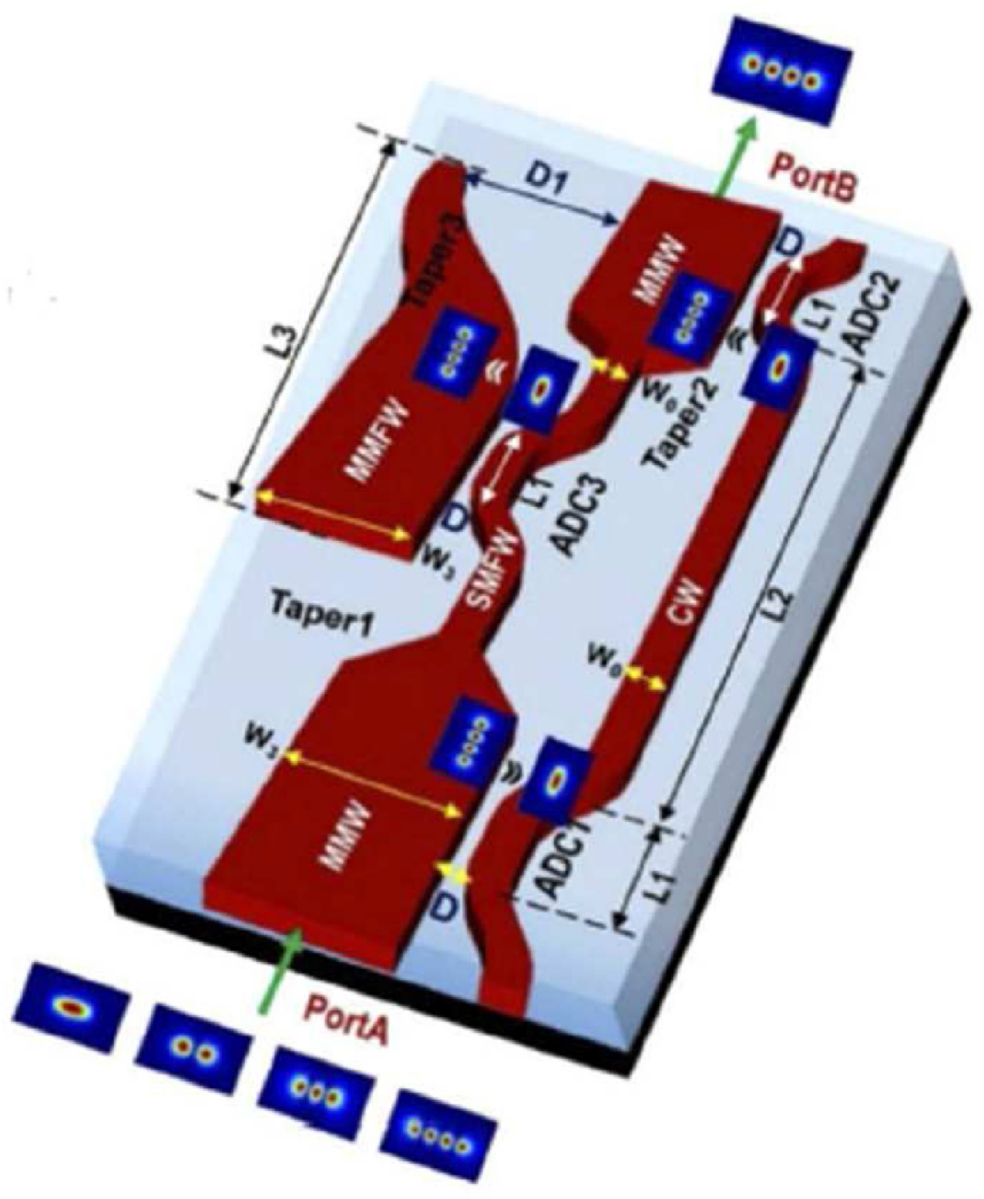
Diagram of the TE_3_ mode pass filter, Reprinted with permission from [36] © The Optical Society.

Recently a highly flexible structure has been proposed and experimentally demonstrated using Asymmetric Directional Couplers (ADC) [36]. The unique feature of this design concept is scalability. This design architecture can pass any arbitrary high-order mode and block other undesired modes. In this work, both the three-mode pass filter and four-mode pass filter have been experimentally demonstrated as proof of that concept. Figure 8 is the diagram of a four-mode (TE_3_) pass filter where the multi-mode waveguide (MMW) is designed to support the TE_0_, TE_1_, TE_2_ and TE_3_ modes only. The single-mode filter waveguide (SMFW) supports only the fundamental mode and blocks all other modes. The width of the coupling waveguide (CW) w_0_ and the width of the multi-mode filter waveguide (MMFW) w_2_ are selected as though the phase-matching condition is satisfied. When the TE_3_ mode is launched at Port A the ADC1 couples the TE_3_ mode from MMW to the TE_0_ of the CW and at Port B the ADC2 will couple back the TE_0_ to the TE_3_ mode. When the TE_2_ and the TE_1_ mode are launched at Port A, they cannot propagate through the SMFW and radiate out through the cladding. As the SMFW allows the TE_0_ mode, when it is launched at Port A, it propagates through the SMFW. Then the ADC3 will couple the fundamental mode to the TE_3_ mode of the MMFW and will be blocked at the Taper 3. Thus the design concept passes the high-order mode and blocks other low-order modes. Any high-order mode can be simply filtered similarly using this design by adjusting the width of the CW to satisfy the phase-matching conditions.

**Figure 9:**
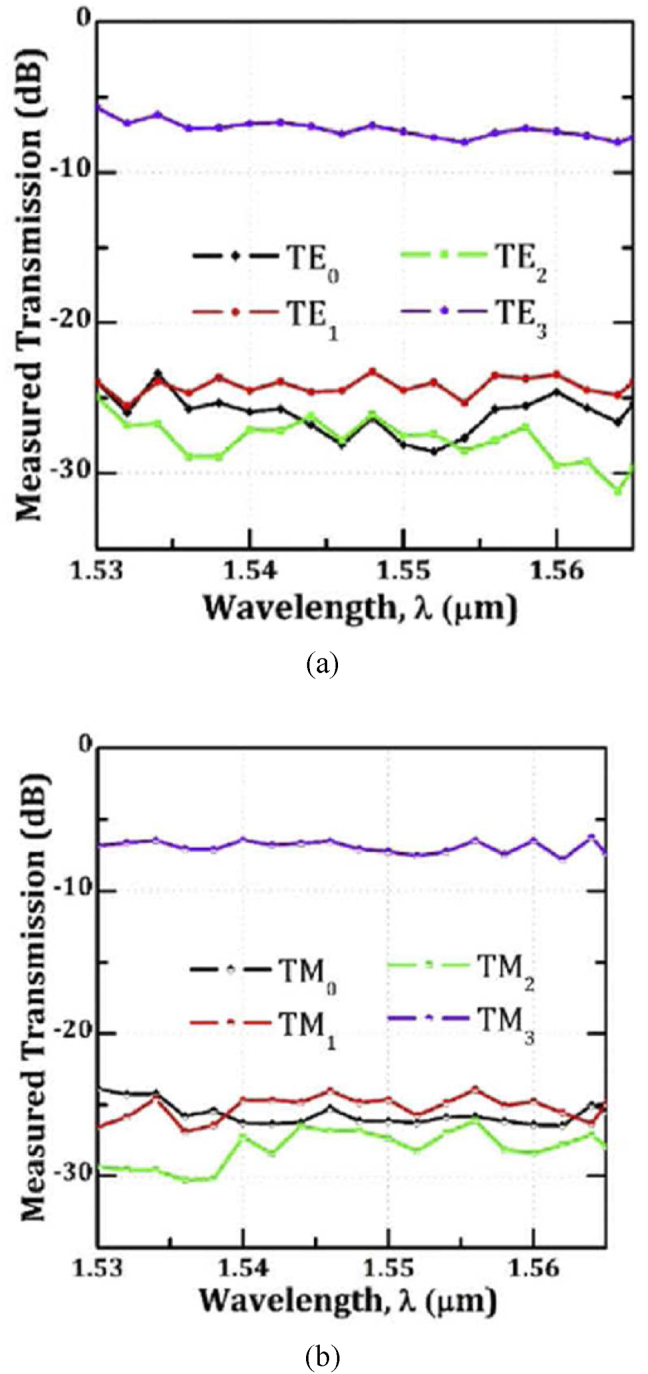
Experimentally measured transmission for (a) the TE and (b) the TM polarization, Reprinted with permission from [36] © The Optical Society.

Figure 9 represents the experimental data of measured transmission spectra for a 4-mode HOM pass filter [36]. These results indicate that this device successfully passes the higher-order mode (TE_3_ or TM_3_ mode) selectively with a small insertion loss. This device is the only device that has passed successfully the three-order mode with lower modal-crosstalk in the waveguide platform.

This design concept is the most promising and effective method of filtering high-order modes. The special features of this device compared to another research are-

✓ High Scalability by simply adjusting the structural parameters
✓ No cascaded structure needed to filter optical modes
✓ Negligible polarization dependencies for the TE and TM polarization
✓ High selectivity
✓ Don't require materials with special optical properties (such as graphene, VO_2_ and hyperbolic material)
✓ High flexibility
✓ Compatible with other PIC platforms

## 4 Findings

Table 1 shows the results including different techniques and fabrication methods used for mode filtering in previous research.

**Table 1:** An overview of research highlights mode filtering.

Reference	Techniques	Fabrication	Results
X. Guan et al. [13]	1D photonic crystal grating	EBL	ER∼ 48 dB, IL∼ 1.80 dB
T. Huang et al. [21]	VO_2_	UV lithography or EBL, Coupled plasma etching	IL ∼ 3.50 dB, ER ∼ 15 dB
Y. Tang et al. [20]	Hyperbolic cladding	Null	Null
K. T. Ahmmed et al. [32]	MZI and Y-junction	Polymer materials (benzocyclobutene) and epoxy OPTOCAST 35O5, conventional microfabrication technology	ER = 37 dB for TE_0_ (1.530–1.565 μm), IL = 0.52 dB
Z. Chang et al. [22]	Graphene-embedded waveguide	Epo-Core and Epo-Clad	IL < 1 dB, ER > 22 dB
Yu He et al. [19]	Sub-wavelength grating-based contra directional coupler	SOI wafer, EBL (Vistec, EBPG5200^+^), inductively coupled plasma etching	ER = 21 dB for TE_0_, ER = 25.60 dB for TE_1_IL < 1.40 dB
Min Teng et al. [35]	MMI and Y-Junction	SOI platform (Vistec VB6-UHR-EWF electron beam lithography)	15 dB ER, IL < 1.50 dB
Chunlei Sun et al. [33]	MZI with Heater and Y-Junction	SOI platform (ultraviolet lithography and inductively coupled plasma etching)	IL ∼3 dB, ∼18 dB ER
P. Xing et al. [23]	GOS	EBL	IL = 2 dB, ER = 310 dBcm^−1^
Quandong Huang et al. [17]	LPG in FMW	Epo-core, Epo-clad	IL > 1.70 dB, ER 10 dB
Guoqing You et al. [24]	Non-linear DBS optimization algorithm	SOI platform with a one-step etching process	IL 0.26 dB, ER = 24.50 dB in 1500–1600 nm for TE_0_
Z. Xing et al. [25]	Graphene nanoribbons	CMP, EBL	TE_1_ to TE_0_ ER∼9.19 and IL∼9.90 dB, TE_2_ to TE_1_ ER∼5.37 and IL∼ 8.40 dB, TE_2_ to TE_0_ ER∼6.44 dB
W. Jiang et al. [18]	Cascaded plasmonic BSWGs	PECVD and RIE	ER∼ 26.40 dB (4-cascaded BSWGs), IL∼0.63 dB
K. T. Ahmmed et al. [36]	ADC	Polymer materials Epo-Core and Epo-Clad (micro resist technology GmbH)	ER > 20 dB in the C-band, IL < 0.50 dB, high selectivity, high scalability, high design flexibility

ER = Extinction ratio, IL = Insertion loss, BCB = benzocyclobutene, GOS = graphene-on-silicon, EBL = electron beam lithography, CMP = Chemical mechanical polishing, SNR = Signal to noise ratio, PECVD = Plasma enhanced chemical vapour deposition, RIE = Reactive ion etching, ADC = Asymmetric directional coupler.

## 5 Conclusion

This review paper presents a brief overview of the recent developments in the designs of the mode filtering techniques in optical fiber communication. This paper also provides a brief knowledge of the concept and the background of the mode filtering. From the results of different researches, it is clear that the use of mode filter increases both the spectral efficiency and transmission capacity. Some techniques of HOM pass filters discussed above have been proposed on some materials with special optical properties which are not available. These techniques can be studied with practically available materials. Designing HOM filter is not the only challenge here but also designing a HOM filter with flexible operation and high scalability is also a challenge. Moreover, in this field, the main demand is to make a device with versatility and high operational bandwidth which can be used commercially to meet the demand of data traffic in future decades. This paper is a summary of the main recent developments in mode filtering along with the performance which gives a direction to future research to solve the capacity crunch.

## Declarations

### Author contribution statement

All authors listed have significantly contributed to the development and the writing of this article.

### Funding statement

This work was supported by the Research and Publication Cell, University of Chittagong.

### Data availability statement

Data included in article/supp. material/referenced in article.

### Declaration of interests statement

The authors declare no conflict of interest.

### Additional information

No additional information is available for this paper.
